# A new species of *Eustigma* (Hamamelidaceae) from Hon Ba Nature Reserve, Vietnam

**DOI:** 10.3897/phytokeys.65.6726

**Published:** 2016-06-15

**Authors:** Hironori Toyama, Shuichiro Tagane, Van Son Dang, Hop Tran, Hidetoshi Nagamasu, Akiyo Naiki, Tetsukazu Yahara

**Affiliations:** 1 Center for Asian Conservation Ecology, Kyushu University, 744 Motooka, Fukuoka, 819-0395, Japan; 2 National Herbarium of Vietnam (VNM), Institute of Tropical Biology, Vietnam Academy of Sciences and Technology, 85.Tran Quoc Toan Str, Dist 3, Ho Chi Minh City, Vietnam; 3 University of Science Ho Chi Minh City, 227 Nguyen Van Cu Street, District 5, Ho Chi Minh City, Vietnam; 4 The Kyoto UniversityMuseum, Kyoto University, Yoshida Honmachi, Sakyo-ku, Kyoto, 606-8501, Japan; 5 Iriomote Station, Tropical Biosphere Research Center, University of the Ryukyus, 870 Uehara, Taketomi-cho, Yaeyama-gun, Okinawa, 907-1541, Japan

**Keywords:** DNA barcoding, flora, Indochina, *matK*, *rbcL*, taxonomy

## Abstract

A new species of Hamamelidaceae, *Eustigma honbaense* H.Toyama, Tagane & V.S.Dang, **sp. nov.**, is described from Hon Ba Nature Reserve, Vietnam. This species is similar to *Eustigma oblongifolium* Gardner & Champ., but differs from it in having entire leaves, longer infructescences, capsules with a longer apical part and seeds with a larger hilum. A description, preliminary conservation assessment, illustration and photographs of the new species are provided, as well as an updated key to the genus *Eustigma*.

## Introduction

*Eustigma* Gardner & Champ. (Gardner 1849) is a small genus of the Hamamelidaceae, distinguished from other genera by small auriculate petals and the greatly enlarged stigma (Harms 1930; Zhang et al. 2003). Currently, three species are known in the genus: *Eustigma balansae* Oliv., *Eustigma lenticellatum* C.Y.Wu and *Eustigma oblongifolium* Gardner & Champ., distributed in mainland China, Laos, Taiwan and Vietnam, among which *Eustigma balansae* is the only species recorded in Vietnam (Tardieu 1965; Hsieh 1993; Hô 2003; Zhang et al. 2003; Newman et al. 2007).

**Figure 1.**
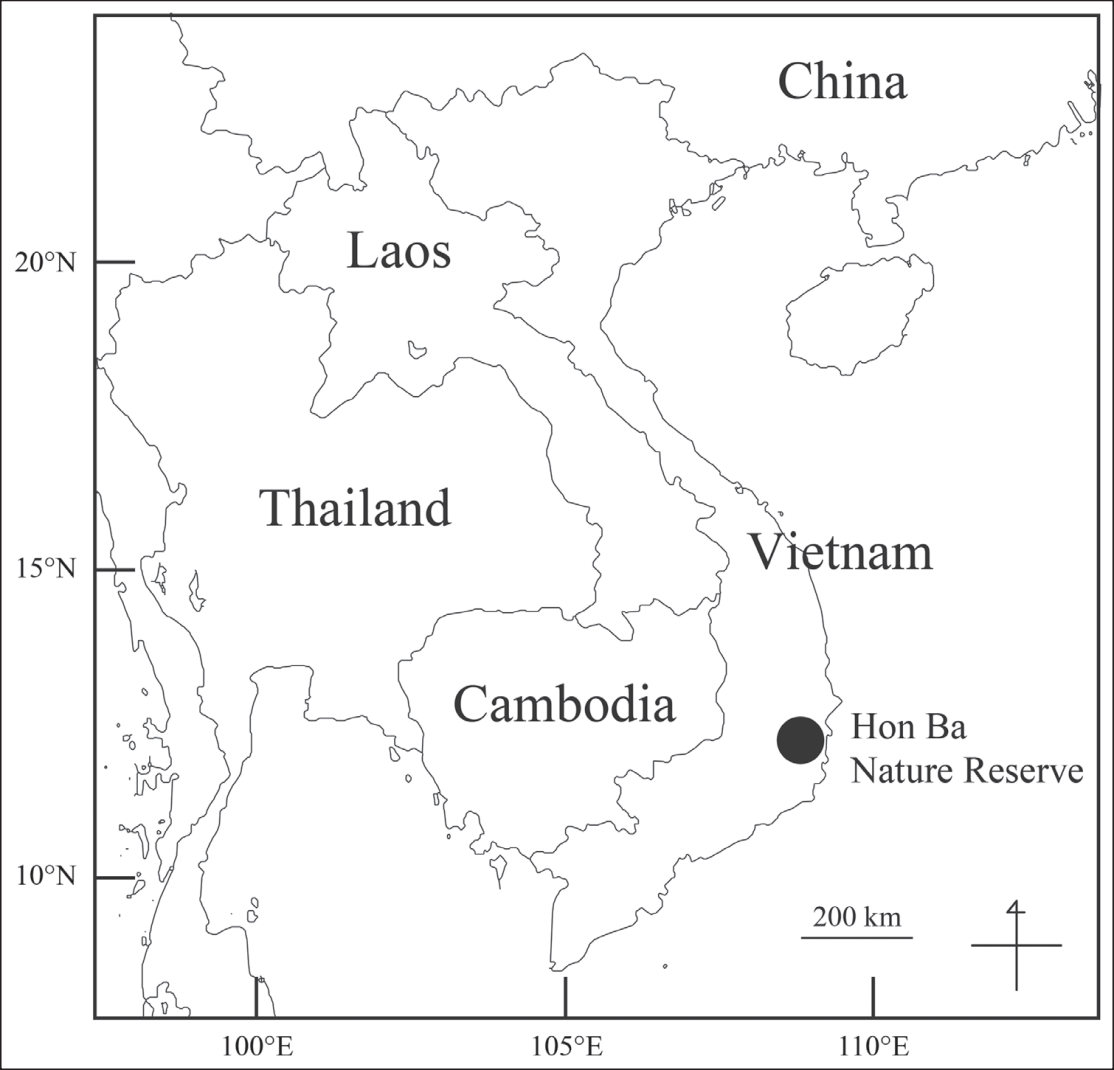
Location of Hon Ba Nature Reserve in Vietnam.

From 2013 to 2014, as part of a collaborative programme to document the biodiversity and ecology of Southeast Asia, Kyushu University
(FU) together with Institute of Tropical Biology, Vietnam (VNM) carried out botanical field surveys in Hon Ba Nature Reserve, in South Vietnam (Figure 1) and found a species of *Eustigma* that was distinct from any of the three known species. Here, we describe this plant as a new species, *Eustigma honbaense* H.Toyama, Tagane & V.S.Dang, and place the species within the wider generic context by providing an updated identification key to all known species of *Eustigma*. Our conclusion is based on observations of specimens in the herbaria, BKF, E, HN, K, KAG, KYO, L, P, TI, and VNM, and specimen images on the website of JSTOR Global Plants (https://plants.jstor.org/). We also provide DNA sequences of two DNA barcode regions; the partial genes for the large subunit ribulose-1,5-bisphosphate carboxylase oxygenase (*rbcL*) and maturase K (*matK*) (CBOL Plant Working Group 2009); established protocols were used to determine the sequences of these regions (Kress et al. 2009; Dunning and Savolainen 2010).

## Taxonomy

### Eustigma honbaense

Taxon classificationPlantaeSaxifragalesHamamelidaceae

H.Toyama, Tagane & V.S.Dang sp. nov.

urn:lsid:ipni.org:names:77155501-1

Figs 2 , 3 , 4

#### Diagnosis.

This species is similar to *Eustigma oblongifolium* Gardner & Champ., but distinguished from that species by having entire elliptic to oblong leaves (vs. partly dentate oblong-lanceolate leaves), infructescences 5–10.3 cm long (vs. 3–5 cm long), apical part (above tepal scar) of capsules 6.5–9 mm long (vs. 3–5 mm long) and seed hilum 4 mm long and 3 mm wide for bigger side (vs. 2–3 mm long and 1–1.5 mm wide).

#### Type.

VIETNAM. Khanh Hoa Province, Hon Ba Nature Reserve, evergreen forest margin along streamside, 401 m alt., 12°06.57'N, 108°59.21'E (DDM), 14 July 2014, *S. Tagane, H. Kanemitsu, V.S. Dang*, *H. Tran*, *X.N. Loi, N.D. Thach*, *N. Dinh & H.N.P. Hieu V1586* (holotype: KYO!; isotypes: FU!, VNM!, the herbarium of Hon Ba Nature Reserve!).

#### Description.

Trees 8–10 m tall. Branches yellow-green when young, turning brown when mature; indumentum sparsely to densely brown stellate hairy when young (denser on the uppermost branch), glabrescent; lenticels more distinct on young branches when dry. Terminal buds naked, narrowly ovoid, ca. 5 mm long, ca. 2 mm wide, having 2 opposite stipules incompletely covering young leaves, linear, ca. 4.5 mm long, ca. 0.5 mm wide, brown stellate hairy. Axillary buds scaled, narrowly ovoid, ca. 3.5 mm long, ca. 1.2 mm wide; bud scales 2, opposite, incompletely covering young leaves, narrowly ovate-oblong, 3–4 mm long, 0.5–1 mm wide, densely brown stellate hairy. Leaf blades elliptic to oblong, 6.5–21.5 cm long, 2.2–8.5 cm wide, coriaceous, lustrous and glabrous on both surfaces except veins below, base obtuse to cuneate, apex acuminate to rounded, margin entire; midribs sunken and glabrous above, prominent and sparsely brown stellate hairy below; secondary veins 5–9 pairs, slightly sunken and glabrous above, prominent and sparsely brown stellate hairy below; tertiary veins reticulate, slightly sunken and glabrous above, prominent and sparsely brown stellate hairy below; petiole 9–14 mm long, sparsely brown stellate hairy; stipules 2, opposite, caducous, linear, 5–8 mm long, 0.5–1 mm wide, brown stellate hairy. Flowers not seen. Infructescences terminal and axillary, racemose, 5–10.3 cm long; peduncles 1.2–4.5 cm long, brown stellate hairy, with 0–2 basal leaves; basal bracts not seen; bracts and bracteoles caducous, narrowly ovate, 1.8–3 mm long, 0.5–1 mm wide (but see note), densely brown stellate hairy; fruiting pedicels 5–8 mm long, densely brown stellate hairy. Floral cups in young fruits, turbinate, 2–3 mm in diameter, densely brown stellate hairy; ovary 2-locular; ovules 1 per locule. Capsules ovoid-globular, 12–16 mm long, 9–10 mm in diameter, woody, dehiscing loculicidally by two 2-lobed valves, sparsely lenticellate, sparsely brown stellate hairy, the length above tepal scar 6.5–9 mm; endocarp loose from woody exocarp. Seeds 2 per capsule, narrowly ovoid, ca. 10 mm long, ca. 4.5 mm wide, ca. 3 mm thick, brownish black, smooth, hilum ca. 4 mm long, ca. 3 mm wide for bigger side (the basal side on the placenta).

#### Other specimen examined.

VIETNAM. Khanh Hoa Province, Hon Ba Nature Reserve, evergreen forest margin along streamside, 393 m alt., 12°06.51'N, 108°59.26'E (DDM), 22 November 2014, *H. Toyama, S. Tagane, V.S. Dang, H. Nagamasu, A. Naiki, H. Tran, C.J. Yang, H.Q. Cuong & H.N.P. Hieu V1975* (FU!, VNM!, the herbarium of Hon Ba Nature Reserve!).

#### Distribution and habit.

This species is only known from Hon Ba Nature Reserve of southern Vietnam. We found only three individuals in the evergreen forest along a stream at ca. 400 m alt. The flora of this area is reported in Choudhary et al. (2012), Schuiteman et al. (2013) and Tagane et al. (2015a, b).

#### Phenology.

Flowering season is unknown. Immature fruits and capsules were observed in July and November.

#### Etymology.

*Eustigma honbaense* is named after the type locality, Hon Ba Nature Reserve.

#### Preliminary conservation status.

*Eustigma honbaense* was collected from a single locality in the Hon Ba Nature Reserve, where only three individuals (one reproductive tree and two young trees) were found in evergreen forest along a streamside. The forest around the habitat was frequently logged and disturbed. Therefore, this species is assessed as Critically Endangered (CR) using the criterion D of the Red List Categories (IUCN 2012), although more individuals could be discovered by more thorough surveys.

#### Note.

*Eustigma honbaense* has terminal naked buds and axillary scaled buds that are in contrast with the description of *Eustigma* having naked-buds as given in the Flora of China (Zhang et al. 2003). The naked bud is covered either with immature leaves that develop to foliage leaves or with their stipules, while the scaled bud is covered with cataphylls or stipules that are highly modified to protect the shoot tip (Nitta and Ohsawa 1998). The terminal buds of *Eustigma honbaense* are classified as naked buds because they are incompletely covered by 2 stipules (Fig. 4A, B, E & F), while the axillary buds are classified as scaled buds because they are covered by 2 cataphylls that are subsequently shed or remained at the tip of previous shoot without further growth (Fig. 4C, D, E & F). The terminal bud of Fig. 4B shows alternate leaf arrangement from the axillary bud, but same direction is also observed.

**Figure 2.**
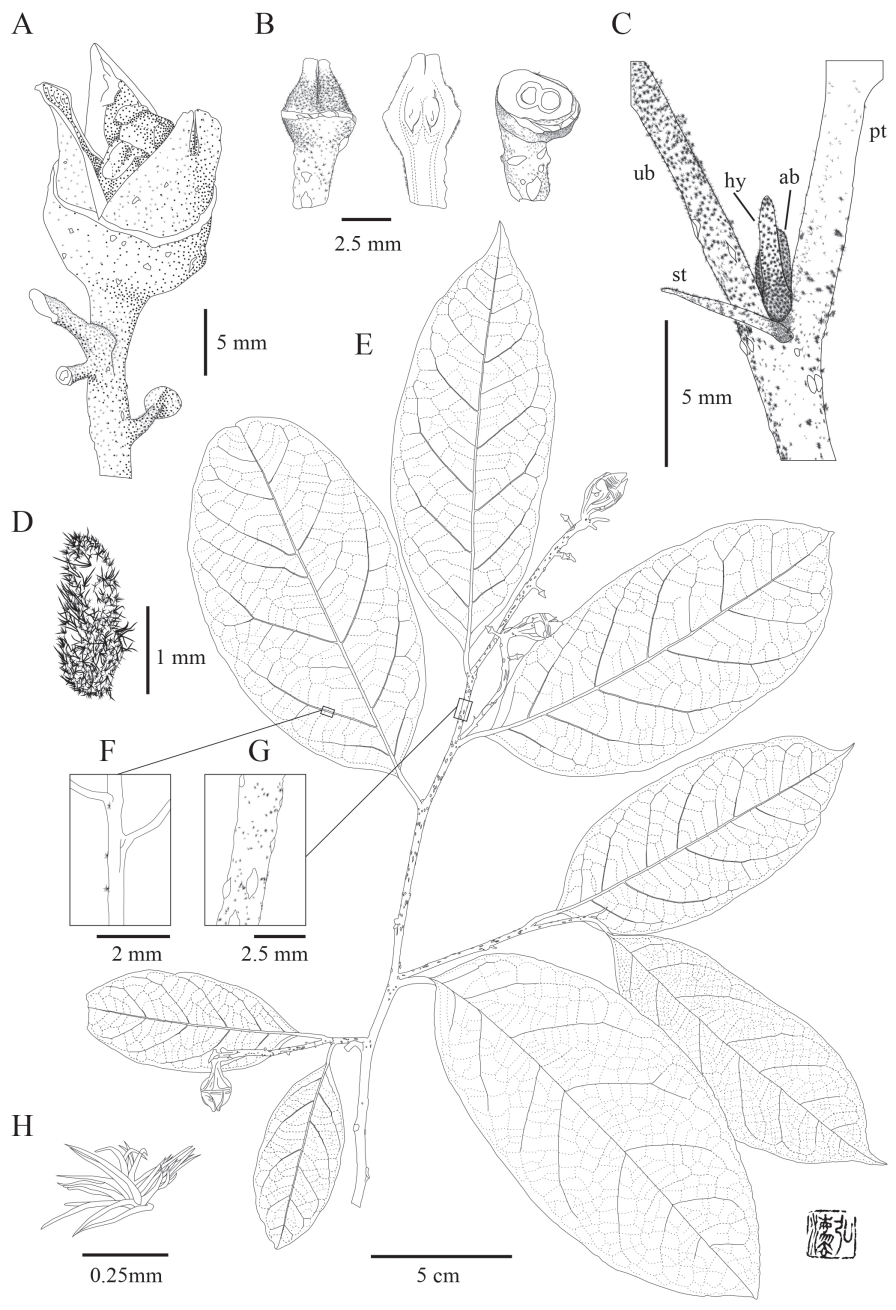
*Eustigma honbaense* H.Toyama, Tagane & V.S.Dang, sp. nov. **A** capsule; **B** immature fruits (lateral view, longitudinal and transverse sections from left) **C**, axillary bud (ab), bud scale (bs), petiole (pt) and stipule (st) and uppermost branch (ub) **D** bracteole **E** fruiting branch **F** vein with stellate hair **G** young branch **H** stellate hair on bud. [**A–H** from *Tagane et al. V1586*, KYO. Drawn by H. Toyama.]

**Figure 3.**
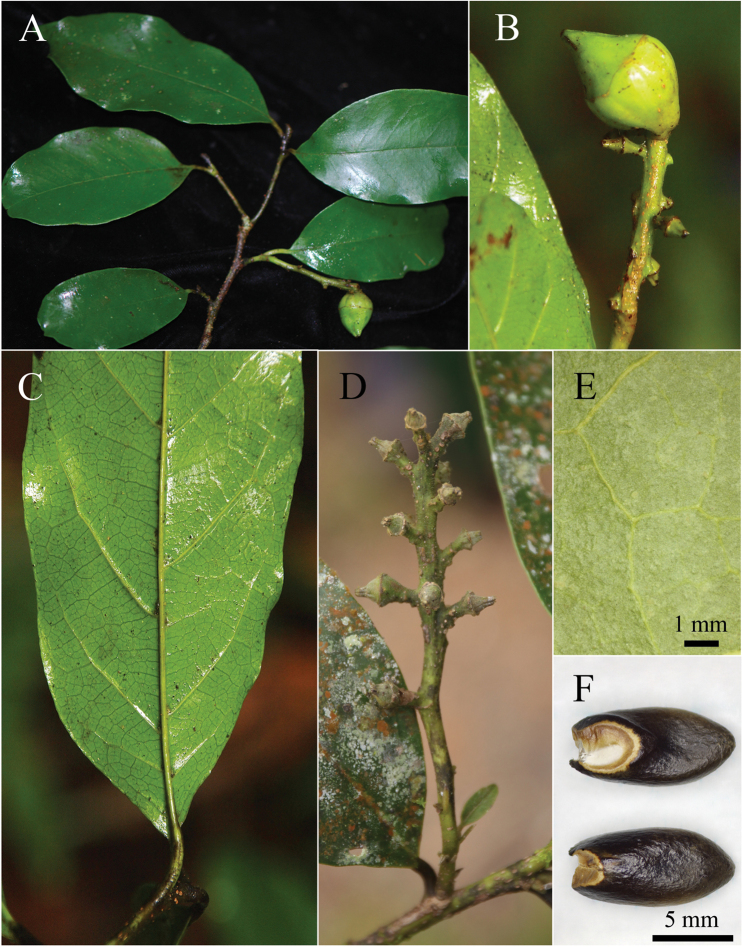
*Eustigma honbaense* H.Toyama, Tagane & V.S.Dang, sp. nov. **A** branch with infructescence **B** fruits; **C** abaxial surface of leaf **D** young infructescence **E** lamina showing glabrous abaxial surface **F** seeds with a large hilum showing basal side on the placenta (upper), and apical side (lower). [**A–D** photographed on 14 July 2014, **E** & **F** from *Tagane et al. V1586*, KYO.]

**Figure 4.**
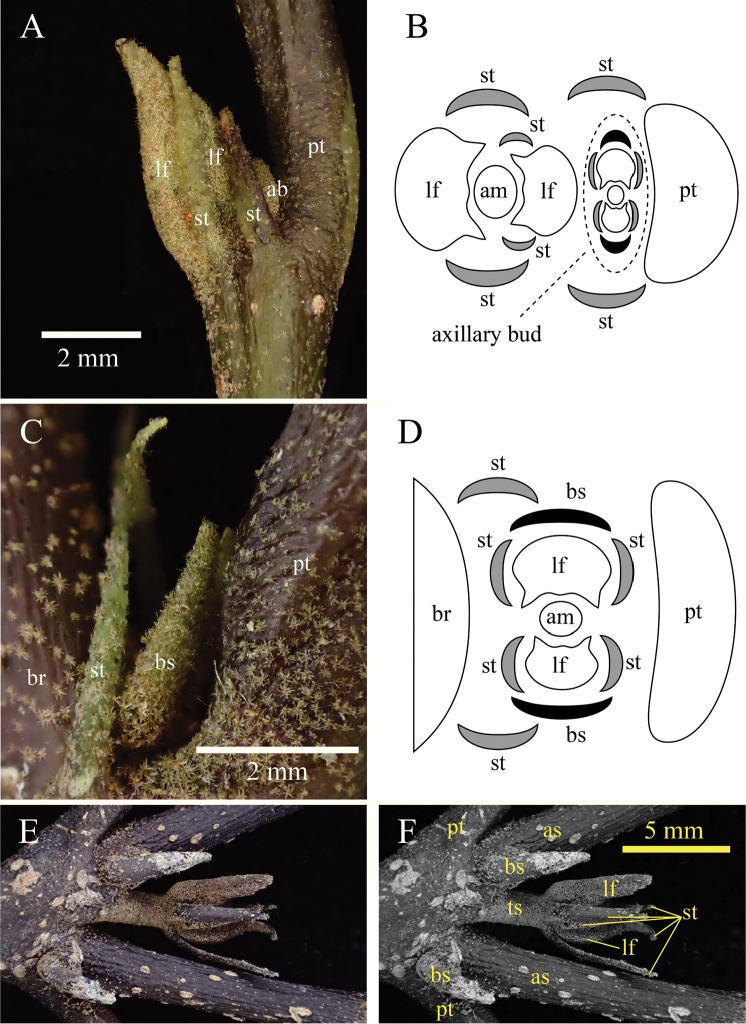
Terminal and axillary bud of *Eustigma honbaense* H.Toyama, Tagane & V.S.Dang, sp. nov. **A** terminal and axillary bud **B** schematic diagram of transverse section of Fig. 4A **C** axillary bud **D** schematic diagram of transverse section of Fig. 4C **E** elongated terminal and axillary shoot **F** grayscale image of Fig. 4E. Abbreviations are as follows: axillary bud (ab), apical meristem (am), axillary shoot (as), bud scale (bs), branch (br), leaf (lf), petiole (pt), stipule (st), terminal shoot (ts). [**A** & **C** photographed on 22 November 2014, **E** & **F** from *Toyama et al. V1975*, FU.]

The description of bracts and bracteoles is insufficient because most of the bracts and bracteoles had been shed. *Eustigma oblongifolium* and *Eustigma lenticellatum* have 3-bracteate flowers (Gardner 1849; Hsieh 1993; Wu 1977), while *Eustigma balansae* has 2-bracteate flowers (Oliver 1891; Tardieu 1965). This could be a key trait to distinguish between species. Further collections of *Eustigma honbaense* are needed.

The *matK* sequences between *Eustigma honbaense* and *Eustigma oblongifolium* (AF013043) differ in 6 bases of the 781 total. The *rbcL* and *matK* sequences between *Eustigma honbaense* and *Eustigma balansae* (*rbcL*: HQ415214, *matK*: HQ415379) differ in 1 base of the 517 total and 5 bases of the 761 total, respectively.

#### Genebank accession No.

*Tagane et al. V1586*: LC005200 (*rbcL*), LC005201 (*matK*).

### An updated key to the species of *Eustigma*

**Table:** 

1	Leaf lamina brown tomentose with stellate hairs on abaxial surface	***Eustigma balansae***
–	Leaf lamina glabrescent except along veins on abaxial surface	**2**
2	Capsules densely lenticellate	***Eustigma lenticellatum***
–	Capsules smooth or sparsely lenticellate	**3**
3	Leaves oblong-lanceolate, margin sometimes dentate towards the apex; infructescences 3–5 cm long, capsule glabrous	***Eustigma oblongifolium***
–	Leaves elliptic to oblong, margin entire, infructescences 5–10.3 cm long, capsule sparsely brown stellate hairy	***Eustigma honbaense***

## Supplementary Material

XML Treatment for Eustigma honbaense
